# *ZmG6PDH1* in glucose-6-phosphate dehydrogenase family enhances cold stress tolerance in maize

**DOI:** 10.3389/fpls.2023.1116237

**Published:** 2023-03-09

**Authors:** Xin Li, Quan Cai, Tao Yu, Shujun Li, Sinan Li, Yunlong Li, Yan Sun, Honglei Ren, Jiajia Zhang, Ying Zhao, Jianguo Zhang, Yuhu Zuo

**Affiliations:** ^1^ National Coarse Cereals Engineering Research Center, Heilongjiang Provincial Key Laboratory of Crop-Pest Interaction Biology and Ecological Control, Heilongjiang Bayi Agricultural University, Daqing, Heilongjiang, China; ^2^ Key Lab of Maize Genetics and Breeding, Heilongjiang Academy of Agricultural Sciences, Harbin, Heilongjiang, China; ^3^ College of Agriculture, Northeast Agricultural University, Harbin, Heilongjiang, China

**Keywords:** *ZmG6PDH1*, enzyme activity, expression, cold stress, CRISPR/Cas9, maize (*Zea mays* L.)

## Abstract

Glucose-6-phosphate dehydrogenase (G6PDH) is a key enzyme in the pentose phosphate pathway responsible for the generation of nicotinamide adenine dinucleotide phosphate (NADPH), thereby playing a central role in facilitating cellular responses to stress and maintaining redox homeostasis. This study aimed to characterize five *G6PDH* gene family members in maize. The classification of these ZmG6PDHs into plastidic and cytosolic isoforms was enabled by phylogenetic and transit peptide predictive analyses and confirmed by subcellular localization imaging analyses using maize mesophyll protoplasts. These *ZmG6PDH* genes exhibited distinctive expression patterns across tissues and developmental stages. Exposure to stressors, including cold, osmotic stress, salinity, and alkaline conditions, also significantly affected the expression and activity of the *ZmG6PDHs*, with particularly high expression of a cytosolic isoform (ZmG6PDH1) in response to cold stress and closely correlated with G6PDH enzymatic activity, suggesting that it may play a central role in shaping responses to cold conditions. CRISPR/Cas9-mediated knockout of *ZmG6PDH1* on the B73 background led to enhanced cold stress sensitivity. Significant changes in the redox status of the NADPH, ascorbic acid (ASA), and glutathione (GSH) pools were observed after exposure of the *zmg6pdh1* mutants to cold stress, with this disrupted redox balance contributing to increased production of reactive oxygen species and resultant cellular damage and death. Overall, these results highlight the importance of cytosolic *ZmG6PDH1* in supporting maize resistance to cold stress, at least in part by producing NADPH that can be used by the ASA-GSH cycle to mitigate cold-induced oxidative damage.

## Introduction

Glucose-6-phosphate dehydrogenase (G6PDH, EC 1.1.1.49) is a ubiquitously expressed enzyme responsible for catalyzing the rate-limiting first step of the pentose phosphate pathway (PPP) in which b-D-glucose-6-phosphate (G6P) is oxidized to 6-phosphoglucono-d-lactone and oxidized nicotinamide adenine dinucleotide phosphate (NADP^+^) is reduced to yield NADPH (Chen et al., 2022). NADPH, produced through PPP-mediated oxidation, functions as a reducing agent essential for redox homeostasis and lipid biosynthesis (Esposito, 2016). In parallel, the non-oxidative arm of the PPP is responsible for generating a range of metabolic intermediates, including ribose-5-phosphate, which is required for nucleotide biosynthesis, and erythrose 4-phosphate, which serves as a precursor molecule for aromatic amino acids and coenzymes (Corpas et al., 2021).

*G6PDH* gene family members have been characterized in many plant species, including soybean (Zhao et al., 2020), strawberry (Zhang et al., 2020), wheat (Tian et al., 2021), tomato (Landi et al., 2016), barley (Zhao et al., 2015), and *Arabidopsis* (Yang et al., 2019; Linnenbrügger et al., 2022), where they function as critical regulators of growth and development. The different G6PDH isoforms are classified according to their subcellular localizations, with each regulated by distinct mechanisms and playing different roles in plant metabolic activity (Feng et al., 2020; Landi et al., 2021). Plastid G6PDH isoforms include P1-G6PDH, which is similar to algal forms of this enzyme and is only expressed in green tissues, and P2-G6PDH, primarily expressed in roots and heterotrophic tissue types (Cardi et al., 2013; Preiser et al., 2019). Cytosolic G6PDH isoforms are estimated to account for 60-80% of total G6PDH activity measured within plant cells (Scharte et al., 2009; Zhang et al., 2020). In *Arabidopsis*, the cytosolic G6PDH knockdown can suppress seed oil accumulation, highlighting a pivotal role for Cy-G6PDH as a regulator of lipid biosynthesis in developing seeds (Ruan et al., 2022).

G6PD activity levels are positively correlated with a range of biotic and abiotic stressors, including fungal pathogen infection, ABA exposure, cell death responses, cold stress, drought, salt stress, nutrient starvation, and aluminum toxicity (Feng et al., 2020; Zhao et al., 2020; He et al., 2021; Huang et al., 2022). Scharte et al. demonstrated that G6PDH activity levels were elevated in the resistant *Nicotiana tabacum* Samsun NN cultivar in response to *Phytophthora nicotianae* infection, whereas the same was not true in the susceptible Xanthi cultivar (Scharte et al., 2009). G6PDH enzymatic responses to heavy metal stress in *Phaseolus vulgaris* L. and wheat are reportedly regulated by aluminum or zinc (Van Assche et al., 1988). RNAi studies have confirmed that the G6PDH isoenzyme shapes drought tolerance and flowering in tobacco plants (Begcy et al., 2012). The *A. thaliana* Cy-G6PDH isoform engages in specific regulatory functions resulting from Thr467 phosphorylation mediated by glycogen synthase kinase 3 (ASKa), with this activity possibly associated with a sugar-sensing signal in response to salt stress (Yang et al., 2019). G6PDH transcript levels in *Triticum aestivum L.* exposed to 0.15 M NaCl stress also reportedly rise with time, peaking after 12 h (Nemoto and Sasakuma, 2000). Zhang et al. determined that G6PDH is also a key enzyme in *Oryza sativa* cells in suspension when exposed to salt stress, maintaining redox homeostasis by regulating G6PDH and NAPDH oxidase activity (Zhang et al., 2013). Cold stress represents a severe physiological constraint for plants, negatively affecting both growth rates and development. Several researchers reported the tolerance of G6PDH to cold stress (Yang et al., 2014; Zhang et al., 2014; Landi et al., 2021). *PsG6PDH* overexpression in transgenic tobacco plants increased the induction of cold stress response-related genes, suggesting a role for this enzyme in the coordination of plant responses to low temperature stress (Lin et al., 2005). A remarkable increase of the expression levels of cytosolic and plastidic *G6PDH* has been found in strawberry (*Fragaria ananassa*) exposed to cold stress (Zhang et al., 2020). Cytosolic- and peroxisome-located G6PDHs showed a central role in acclimation to cold stress at various growth stages of barley (Hordeum vulgare) and *Arabidopsis thalian*a (Tian et al., 2021). While G6PDH activity is thus known to be central to the induction of plant responses to abiotic stressors, the specific relationships between its enzymatic reactions and stress tolerance are not fully understood.

The roles of G6PDH isoforms as coordinators of stress response-related activities have been documented in many plants, but little is known about their function in maize (*Zea mays* L.). Maize is the most widely produced crop in the world and is a significant component of animal feed and raw material used in industrial applications (Sheoran et al., 2021). Five maize G6PDH (ZmG6PDH) gene family members were characterized in this study. Transit peptide analyses were initially used to predict the localization of these ZmG6PDHs within cells, further confirmed by transient expression of GFP-tagged ZmG6PDHs in maize protoplasts. The transcriptional profiles of *ZmG6PDH* were further analyzed using high-throughput sequencing and qPCR in multiple organs and response to various abiotic stressors. The results showed that the transcription of one cytosolic isoform (*ZmG6PDH1*) was sensitive to cold stress and was correlated with G6PDH enzyme activity levels. Knockout of *ZmG6PDH1* reduced the tolerance of transgenic maize seedlings to cold stress, with corresponding reductions in the NADPH/NADP^+^, GSH/GSSG (reduced/oxidized glutathione), and ASA/DHA (ascorbic acid/dehydroascorbate) ratios, together with higher levels of reactive oxygen species (ROS) production. These findings suggest that these *ZmG6PDHs* may be important regulators of plant growth and stress response activity, with cytosolic *ZmG6PDH1* being the primary isoform responsible for regulating cellular redox pools and mitigating oxidative stress.

## Materials and methods

### Maize *G6PDH* gene family identification

Maize *G6PDH* isoforms were identified using known *A. thaliana G6PDH* sequences as queries to perform a BLASTP search against the maize genome (http://www.maizesequence.org). Protein sequences for candidate ZmG6PDHs exhibiting >90% sequence identity and an E-value of <10^-10^ were downloaded. Phytozome v13 (https://phytozome-next.jgi.doe.gov/) was then used to acquire details regarding the genetic characteristics of these *ZmG6PDH* family genes, including chromosome location, coding sequence length, and protein length. The ExPASy server (http://expasy.org/) was used to determine the molecular mass and isoelectric points of these proteins, and predictions of transit peptides and subcellular localization were conducted using TargetP 2.0 (http://www.cbs.dtu.dk/services/TargetP/) and CELLO 2.5 (http://cello.life.nctu.edu.tw/) (Yu et al., 2004).

### Evolutionary, synteny, and gene structural analyses

Full-length G6PDH protein sequences from Z. mays (ZmG6PDH1-5), Solanum lycopersicum (SlG6PDH1-4), Setaria italica (SiG6PDH1-5), Triticum aestivum (TaG6PDH1-5), Solanum tuberosum (StG6PDH1-4), Brassica oleracea (BolG6PDH1-5), Phaseolus vulgaris (PvG6PDH1-5), Sorghum bicolor (SbG6PDH1-4), and A. thaliana (AtG6PDH1-6) were utilized to construct a neighbor-joining phylogenetic tree by MEGA 5.0 software with the bootstrap values performed on 1000 replicates (Tamura et al., 2011). These amino acid sequences were aligned with ClustalW with standard settings (gap opening penalty: 10 and gap extension penalty: 0.2) (Larkin et al., 2007). The GSDS database (http://gsds.cbi.pku.edu.cn/index.php) confirmed G6PDH gene structural characteristics by aligning coding regions and associated genomic regions. The NCBI and maize genetics and genomics databases were used to obtain A. thaliana and Z. mays genomic and coding sequences for G6PDHs. Syntenic blocks among the G6PDHs encoded by Z. mays, P. vulgaris, A. thaliana, *S. lycopersicum*, *S. italica*, T. aestivum, S. tuberosum, B. oleracea, and S. bicolor were then established based on the plant genome duplication database (PGDD, http://chibba.agtec.uga.edu/duplication/) (Lee et al., 2012). Gene IDs and other details regarding G6PDHs utilized for these analyses are compiled in **Supplementary Table S1**.

### *ZmG6PDH* promoter analyses

Key *cis*-acting elements in the promoter regions of candidate *ZmG6PDHs* were identified using the maize genetics and genomics database to obtain the region 2.0 kb upstream of the ATG start codon for each of these genes. The PlantCARE database (http://bioinformatics.psb.ugent.be/webtools/plantcare/html/) was then used to predict *cis-*acting elements within these regions, presented using IBS 2.0 (Liu et al., 2015).

### Subcellular localization analyses

The complete coding regions corresponding to the five identified *ZmG6PDHs* from the inbred B73 maize variety were amplified by RT-PCR using high-fidelity KOD*‐*Plus‐DNA polymerase and gene-specific primers (**Supplementary Table S2**). The amplified genes were inserted into the pBI121 vector, containing a GFP tag and a CaMV35S promoter. The resultant *pBI121-ZmG6PDHs::GFP* fusion proteins were transiently transformed into the maize mesophyll protoplasts isolated from leaves of 14-day-old seedlings using polyethylene glycol (PEG)-mediated protoplast transformation technique (Yoo et al., 2007). The localization of these proteins was then visualized using confocal laser-scanning microscopy (LSM 710, Carl Zeiss, Jena, Germany) with respective excitation/emission wavelengths of 488 nm/507-535 nm and 610 nm/650-750 nm for GFP and chlorophyll autofluorescence.

### *ZmG6PDH* expression analyses

Patterns of *ZmG6PDH* gene expression across tissues, including leaves, stems, roots, ears, mature seeds, brace roots, and tassels, were analyzed using high-throughput sequencing data in the Phytozome database. The data were compiled into heatmaps subjected to hierarchical clustering performed with TBtools (Chen et al., 2020), and the values were normalized and subjected to log2 transformation. *ZmG6PDH* expression across different stages of seed development was characterized by extracting total RNA from maize seeds 5, 10, 15, 20, 25, and 30 days after flowering (DAF), with *ZmG6PDH* expression profiles at 5DAF used as a baseline for subsequent expression level changes. *ZmG6PDH* transcriptional profiles in response to different forms of abiotic stress were evaluated by subjecting maize seedlings at the three-leaf stage to salt stress (150 mM NaCl), alkali stress (100 mM NaHCO_3_), osmotic stress (20% w/v PEG [MW: 6000 g/M]), and cold stress (incubation at 4°C). At 0, 3, 6, 12, and 24 h following the initiation of these treatments, total RNA was extracted from the leaves of the seedlings. Levels of *ZmG6PDH* expression in maize leaves under non-stressed conditions served as a baseline for these analyses, while *ZmGAPDH* and *ZmACTIN* were used for normalization (Kong et al., 2013; Zhang et al., 2014). All qPCR assays were performed using three technical and three biological replicates, with relative ZmG6PDH expression levels determined through the 2^-△△ct^ method.

### CRISPR/Cas vector construction and maize transformation

A CRISPR/Cas9 approach was used to generate mutations in the *ZmG6PDH1* coding regions. Two guide RNAs targeting sites in the *ZmG6PDH1* gene were designed with the CRISPR-P 2.0 web tool (Liu et al., 2017) based on the B73 reference genome (**Table S3**), with these guide RNAs then being introduced into the pBUE411 vector (Xing et al., 2014). The resultant pBUE411 binary vector was introduced into *Agrobacterium tumefaciens* strain EHA105. *Agrobacterium*-mediated transformation was conducted with 10-15 DAP immature zygotic embryos (Char et al., 2017). The genome editing results were evaluated by PCR amplification and Sanger sequencing of target regions, and the expression of *ZmG6PDH1* in gene-edited mutants was assessed by qPCR and enzyme activity analyses.

Dry weight values for both mutant and wild-type (WT) plants were assessed on day 5 following treatment at 4°C, with plant height and root length also being recorded. Samples of leaves were collected on day 3 of treatment to analyze biochemical and physiological parameters therein. Experiments were repeated at least three times with 10 to 20 plants, and all images depict representative results. Total chlorophyll, chlorophyll a, and chlorophyll b levels in the top secondary fully expanded leaves were analyzed as reported previously (Wellburn, 1994) using 80% (v/v) acetone. Photochemical efficiency (*F_v_*/*F_m_*) was analyzed with a pulse*-*modulated fluorometer (FMS2, Hansatech, UK), and leaf photosynthetic characteristics (Pn, net photosynthetic rate) were evaluated with a portable open photosynthesis system (Li-6400; Li-Cor, Inc., NE, USA).

### Biochemical and physiological analyses

G6PDH activity was measured using a modified version of a previously reported protocol (Wakao and Benning, 2005). The total reaction volume of the assay was 1 mL containing 3.3 mM MgCl_2_, 50 mM Hepes-Tris (pH 7.8), 0.5 mM NADPNa_2_, 0.5 mM D-glucose-6-phosphate disodium salt, and an appropriate amount of enzyme extracts. The absorbance of the supernatant was read at 340 nm using an ultraviolet spectrophotometer (U3900, Hitachi High-Technologies, Japan). The redox status of the NADPH, ASA, and GSH pools was examined by evaluating the levels of the oxidized (NADP^+^, DHA, GSSG) and reduced (NADPH, ASA, GSH) forms of these intermediates by spectrophotometry, as previously described (Nagalakshmi and Prasad, 2001; Queval and Noctor, 2007).

Superoxide $\left(O_{2}^{-}\right)$ and hydrogen peroxide (H_2_O_2_) levels were also analyzed by spectrophotometry as previously described (Fryer et al., 2002). Thiobarbituric acid-reactive substances (TBARS) and electrolyte leakage (EL) levels were used to assess membrane leakage, as detailed previously (Hodges et al., 1999; Zhao et al., 2019). Leaf tissues (0.5 g) were ground in ice extracted with 10 mL 0.1% (w/v) trichloroacetic acid (TCA), and then the homogenate was centrifuged at 10 000 g for 10 min at 4°C. Supernatants were then collected for analyses of TBARS contents. The total reaction volume of the TBARS assay was 2 mL containing 0.5 mL of the supernatant and 1.5 mL 0.5% (w/v) thiobarbital acid in 15% TCA. The absorbancy of supernatant was read at 532 nm.

ROS scavenging abilities were examined by homogenizing 0.5 g of maize in 0.2 mL extraction buffer (1% PVP, 1.5 mM EDTA, 0.5 mM ASC, K_2_HPO_4_-KH_2_PO_4_, pH 7.0), with homogenates then being centrifuged at 12 000 g for 20 min at 4°C (Nakano and Asada, 1981; Takashi et al., 1997). The supernatants were then analyzed for the activity levels of monodehydro-ascorbate reductase (MDAR), glutathione reductase (GR), glutathione peroxidase (GPX), dehydroascorbate reductase (DHAR), and ascorbate peroxidase (APX) according to the instructions provided with commercial kits (Solarbio, China).

### Statistical analysis

A minimum of three biological replicates were used per experiment. Results are given as means ± SD and were compared with Student’s t-tests using SPSS 22.0. P< 0.05 was the significance threshold.

## Results

### Maize *G6PDH* gene family identification and categorization

Initial analyses of the *Z. mays* genome led to the tentative identification of five genes encoding *G6PDH* isoforms named *ZmG6PDH1-5* (**Table 1**). The full-length coding sequences for these genes were between 1527 and 2748 bp, encoding proteins ranging from 508-915 amino acids in length. Isoelectric points and molecular weights for the candidate ZmG6PDHs encoded by these genes ranged from 6.26-9.22 and 57.63-103.03 kDa, respectively (**Table 1**). TargetP 1.1 and CELLO 2.5 were then utilized to detect putative N-terminal transit peptide (TP) sequences, predicting that ZmG6PDH1 and ZmG6PDH5 were localized in the cytosol while the three other isoforms were expected to localize to the plastid compartment (**Table 1**).

**Table 1:** Basic information of the five maize *G6PDH* genes (*ZmG6PDHs*).

Gene Name	Gene ID	Previous Identifiers	Gene location	ORF length (bp)	Protein length	Isoelectric point	Molecular weight (kDa)	Subcellular localization
*ZmG6PDH1*	Zm00001d003252_T003	GRMZM2G130230_T01	Zm2 37085168-37093582	1527	508	6.31	57.63	Cytoplasm
*ZmG6PDH2*	Zm00001d025015_T002	GRMZM2G177077_T01	Zm10 99381158-99387758	1959	652	7.91	72.81	Chloroplast
*ZmG6PDH3*	Zm00001d047587_T005	GRMZM2G426964_T01	Zm9 134182737-134185295	2748	915	6.26	103.03	Chloroplast
*ZmG6PDH4*	Zm00001d029502_T001	GRMZM2G179521_T01	Zm1 72102454-72121802	1929	642	9.22	71.97	Chloroplast
*ZmG6PDH5*	Zm00001d017119_T003	GRMZM2G031107_T02	Zm5 181440754-181446379	1557	518	6.66	58.62	Cytoplasm

**Figure 1:**
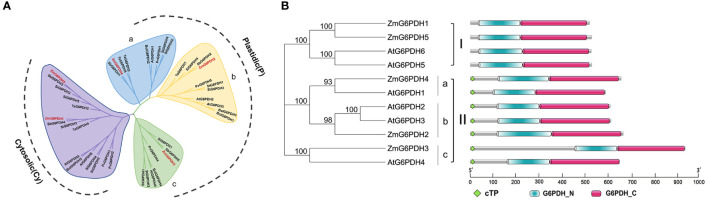
Analysis of the phylogeny and protein domains of ZmG6PDHs. **(A)** Phylogenetic tree based on G6PDH protein sequences from *Z. mays* (red), *A. thaliana*, *P. vulgaris*, *S. lycopersicum*, *S. italica*, *T. aestivum*, *S. tuberosum*, *B. oleracea*, and *S. bicolor*. **(B)** Bi-domain structures of G6PDH proteins from maize and *A. thaliana*.

The evolutionary history and classification of these ZmG6PDHs were explored by aligning their full-length protein sequences with those of homologous G6PDH enzymes encoded by *S. bicolor* (StG6PDH1-4), *S. italica* (SiG6PDH1-5), *S. lycopersicum* (SlG6PDH1-4), *A. thaliana* (AtG6PDH1-6), *T. aestivum* (TaG6PDH1-5), *S. tuberosum* (StG6PDH1-4), *P. vulgaris* (PvG6PDH1-5), and *B. oleracea* (BolG6PDH1-5) to construct a phylogenetic tree (**Figure 1A**). In this analysis, these plant G6PDHs were broadly classified into Clade I (cytosolic isoforms) and Clade II (plastidic isoforms). The cytosolic (Cy) G6PDH isoforms, including ZmG6PDH1 and 5, as well as two *Arabidopsis* Cy-G6PDHs (AtG6PDH5, 6) (Wakao et al., 2008). Members of Clade II were further subdivided into class a (including ZmG6PDH4 and AtG6PDH1), class b (ZmG6PDH2 and AtG6PDH2, 3), and class c (ZmG6PDH3 and the inactive-G6PDH isoform AtG6PDH4). These ZmG6PDHs were closely related to homologs from monocot sorghum plants within individual clusters, consistent with the evolutionary history of these plant lineages and associated G6PDH isoforms.

Structural analyses of the proteins encoded by these five *ZmG6PDHs* revealed the presence of a bi-domain structure akin to that reported for *A. thaliana* G6PDHs, including both an N-terminal NADP^+^-binding domain (PF00479) and a C-terminal G6PD domain (PF02781) (**Figure 1B**). Highly conserved substrate-binding (RIDHYLGKE) and NADP^+^-binding (NEFVIRLQP) motifs were evident in all five ZmG6PDHs (**Figure S1**). Based on the above results, these ZmG6PDHs were classified into three plastidic G6PDH isoforms (ZmG6PDH2, 3, and 4) and two cytosolic G6PDH isoforms (ZmG6PDH1 and 5). These findings highlight the relationships between the specific functions of these G6PDH isoforms and their underlying evolution.

### *ZmG6PDH* syntenic relationships and gene structure analyses

**Figure 2:**
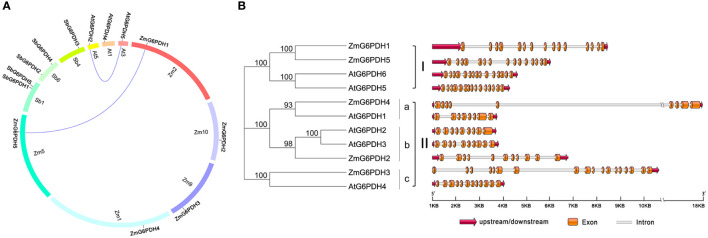
Syntenic and exon-intron structural analyses of genes in the *ZmG6PDH* family. **(A)** *ZmG6PDH* genes were subjected to syntenic analyses together with corresponding genes from *A. thaliana* and *S. bicolor*. Chromosomes are represented by circles, with the collinear regions of the *G6PDH* genes denoted by colored curved regions. **(B)** *AtG6PDH* and *ZmG6PDHs* gene family exon-intron organization. Blue arrows denote untranslated region (UTR) sequences. Colored boxes and gray lines represent exons and introns, respectively.

Over the past three million years, the maize genome has expanded to 2.3 gigabases due to two rounds of genomic duplication mediated by long-terminal-repeat retrotransposon proliferation. Synteny analyses of *G6PDHs* from *Z. mays*, *A. thaliana*, *P. vulgaris*, *S. lycopersicum*, *S. italica*, *T. aestivum*, *S. tuberosum*, *B. oleracea*, and *S. bicolor* were conducted to explore the possible functional roles of these *ZmG6PDHs*. The five *ZmG6PDHs* were scattered across five of the ten maize chromosomes (**Figure 2A**), with one gene per chromosome. *ZmG6PDH1* and *ZmG6PDH5* were identified as a pair of syntenic genes on chromosomes 2 and 5, respectively, consistent with the fact that only two *Arabidopsis G6PDH* genes are syntenic (**Figure 2A**; **Table S4**). Notably, no paralogous or orthologous *GPDH* gene pairs were detected in the other plant species included in this study.

The structural diversity of the *ZmG6PDHs* was further explored by comparing the exon/intron sizes and localizations with those of *AtG6PDHs*, showing that the exon-intron structures of *G6PDHs* in the same clusters were largely similar, particularly regarding the number of exons (**Figure 2B**). For example, *G6PDH* genes in cluster I contained 15 exons, while those in cluster II contained 8-12 exons of nearly identical lengths. These data highlight the conservation of *ZmG6PDH* genes regarding gene sequences and exon-intron organization within phylogenetic groups.

### Identification of regulatory elements in *ZmG6PDH* promoter regions

**Figure 3:**
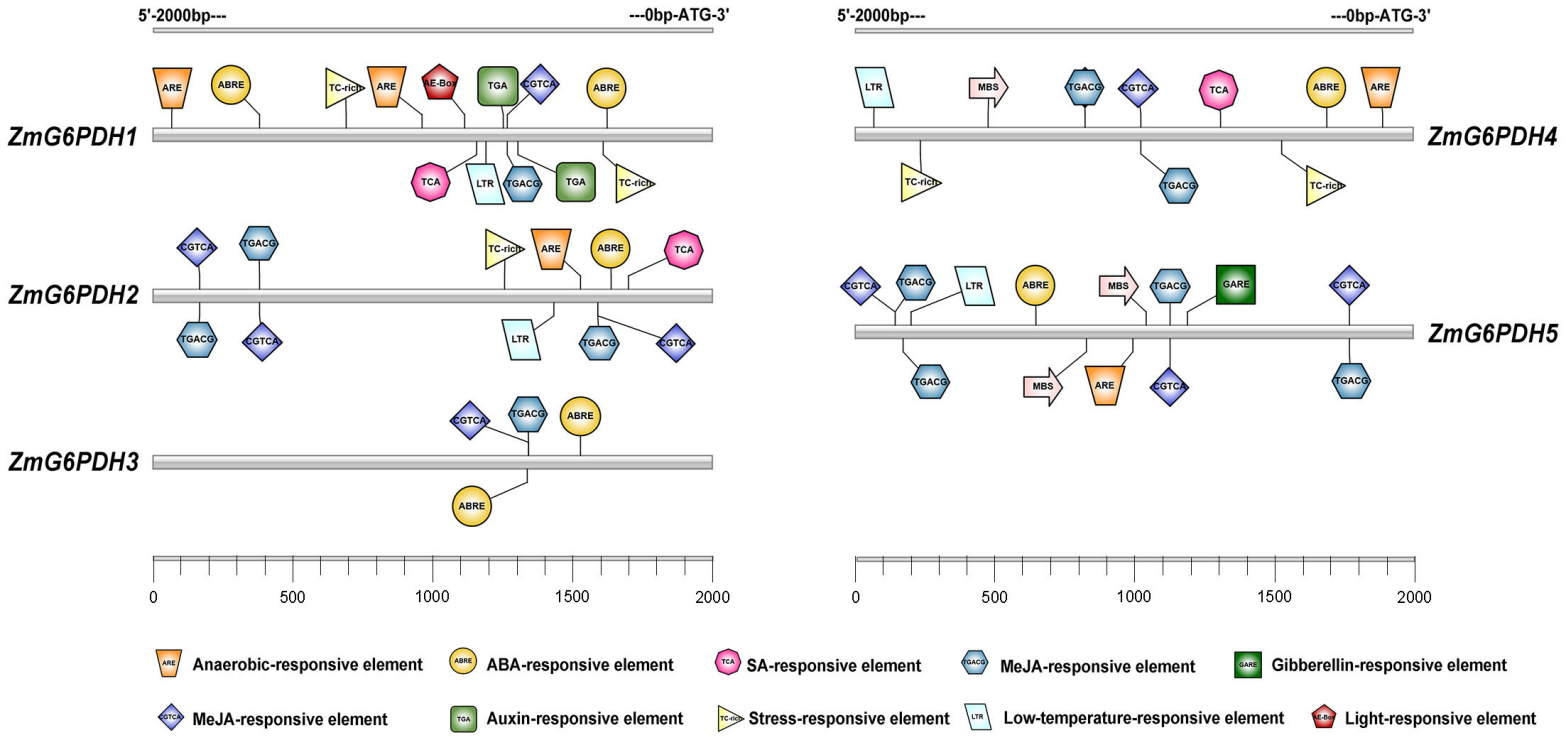
Predicted *cis*-acting elements within the 2.0-kb promoter region upstream of the start codons of *ZmG6PDHs*. Differently colored boxes show relative cis-acting element positions for each *ZmG6PDH*.

Putative *cis*-acting elements that may play a role in the transcriptional regulation of *ZmG6PDHs* were identified by analyzing the region 2.0 kb upstream of the translation start site (ATG) for each of these genes. The majority of these *ZmG6PDHs* contained several stress-responsive *cis-*acting elements, including the anoxic-inducible ARE element, which was present in all genes other than *ZmG6PDH3* (**Figure 3**). The drought response-related MBS element was present in the *ZmG6PDH4* and *ZmG6PDH5* promoter regions, while the stress and defense response-related TC-rich repeat element was observed in the *ZmG6PDH1*, *ZmG6PDH2*, and *ZmG6PDH4* promoters, and the cold-responsive LTR element was observed in the *ZmG6PDH1*, *ZmG6PDH2*, *ZmG6PDH4*, and *ZmG6PDH5* promoters (**Figure 3**). All these *ZmG6PDH* promoters contained at least one hormone-responsive *cis-*acting element, such as the ABA-responsive element (ABRE) and gibberellin-responsive element (GARE).

### Assessment of ZmG6PDH subcellular localization

**Figure 4:**
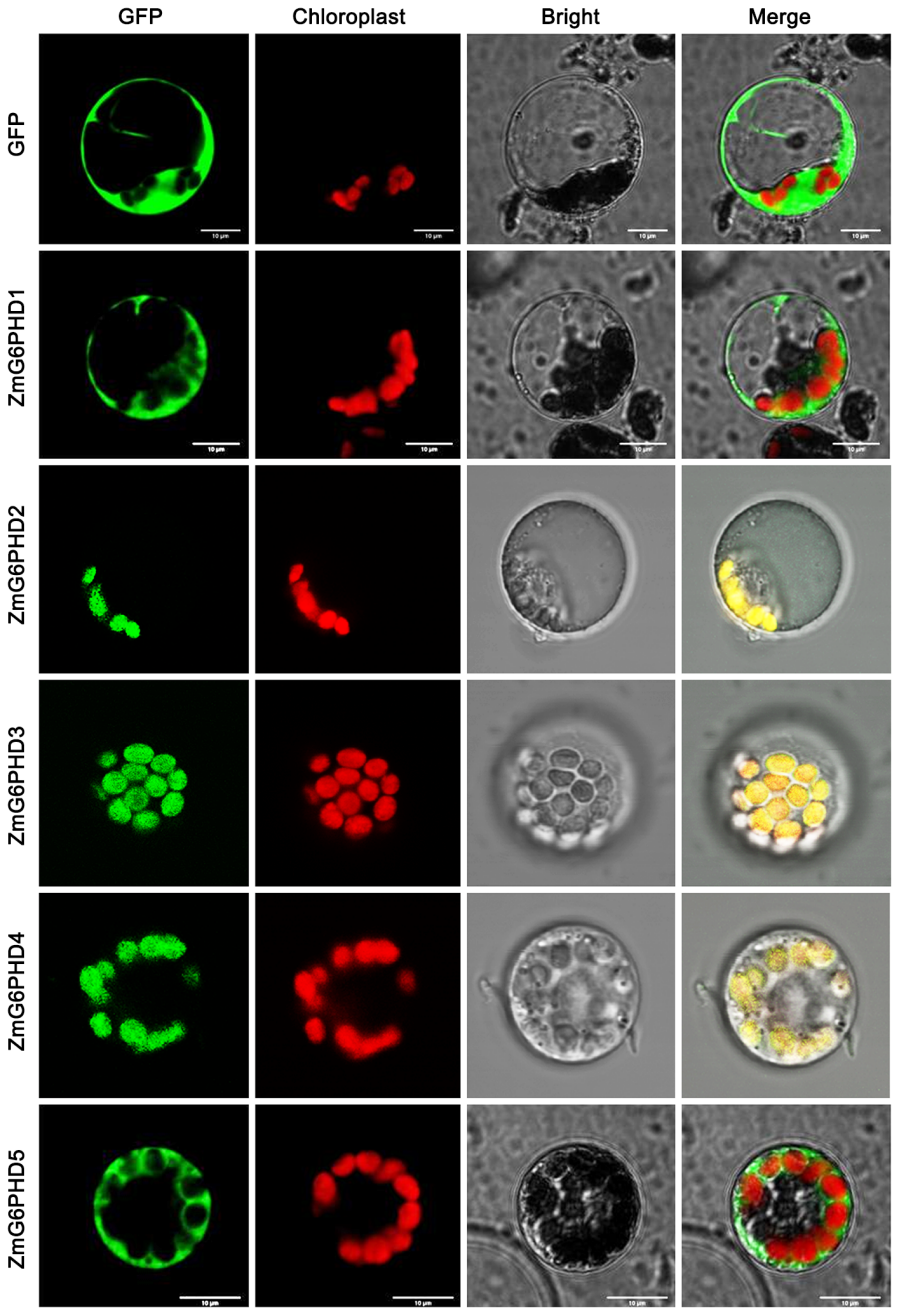
Subcellular localization of ZmG6PDHs analyzed by transient transfection of maize mesophyll protoplasts. Transfection led to the expression of five ZmG6PDH-GFP fusion proteins. Subcellular localization of the ZmG6PDH1, ZmG6PDH2, ZmG6PDH3, ZmG6PDH4, and ZmG6PDH5 proteins was evaluated by confocal microscopy. GFP signals, chloroplast autofluorescence, brightfield images, and merged images are shown in panels 1-4, respectively.

The localization of all five ZmG6PDHs was next verified by cloning their coding sequences; these sequences have been submitted to GenBank under the following accession numbers: *ZmG6PDH1* (ON962526), *ZmG6PDH2* (ON962527), *ZmG6PDH3* (ON962528), *ZmG6PDH4* (ON962529) and *ZmG6PDH5* (ON962530). These cloned coding region sequences were introduced in-frame with an N-terminal sequence encoding GFP. A positive control vector and GFP-tagged ZmG6PDH proteins were transiently transfected into maize mesophyll protoplasts. While free GFP was distributed evenly throughout all cell regions other than the vacuoles and chloroplasts (**Figure 4**), ZmG6PDH2, 3, and 4 specifically localized to the chloroplast compartment, and ZmG6PDH1 and ZmG6PDH5 were only detected in the cytosol (**Figure 4**). These findings were consistent with the predictive analyses described above.

### Analyses of *ZmG6PDHs* expression across different tissues and stages of development

**Figure 5:**
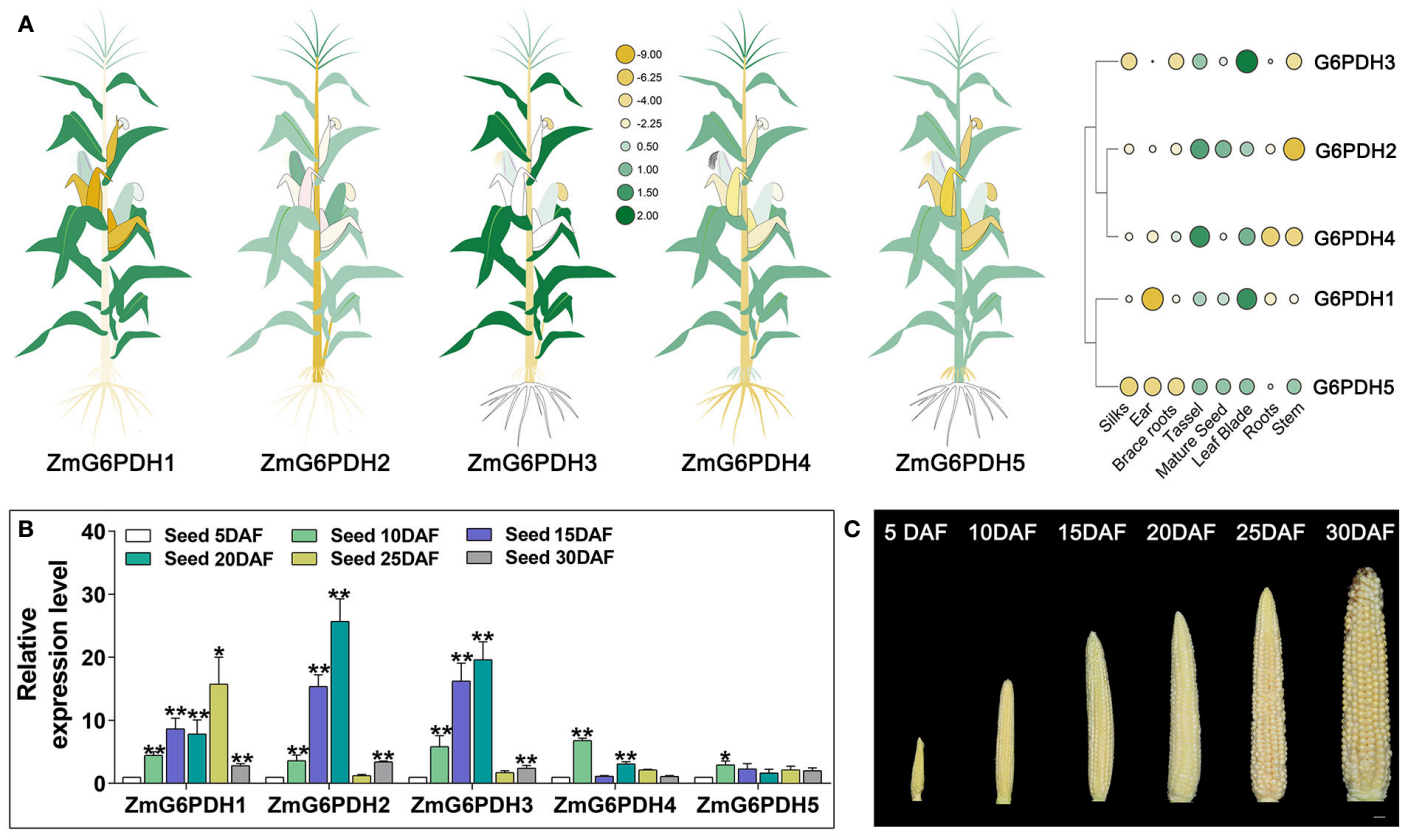
Transcripts of *ZmG6PDHs* in various tissues. **(A)** *ZmG6PDH* transcript levels were investigated in different tissues using the Phytozome database. Heatmap construction was performed using TBtools based on log2 expression levels. In the heatmaps, green and yellow indicate high and low transcription levels, respectively. Larger circles indicate higher levels of transcription. **(B)** Transcriptional profiles and **(C)** physiological phenotypes corresponding to *ZmG6PDHs* at 5, 10, 15, 20, 25, and 30 days after flowering (DAF). The expression of *ZmG6PDHs* in developing seeds at 5 DAF was used as an internal reference. Three biological replicate samples were analyzed per tissue. *P< 0.05, ** P< 0.01; Student’s t-test.

Next, *ZmG6PDH* expression levels were systematically evaluated in multiple tissues and seeds on days 5, 10, 15, 20, 25, and 30 after flowering *via* qPCR. The *ZmG6PDHs* showed tissue-specific expression patterns (**Figure 5A**). *ZmG6PDH1*, *3*, and *5* were primarily detected in leaf blade samples, whereas *ZmG6PDH2* and *ZmG6PDH4* were primarily detected in tassel samples. Most *ZmG6PDHs* were expressed at low levels in stem, ear, silk, and brace root samples. These results suggest that *ZmGPDHs* may play a range of roles in the growth and development of maize plants. When these expression levels were assessed in seeds throughout development, all *ZmG6PDHs* were found to be expressed at relatively high levels during the early-middle stage of development from 10-20 DAF (**Figure 5B**), whereas they were expressed at low levels during later stages of maturation and development at 25-30 DAF except for *ZmG6PDH1* (**Figure 5B**). Maximal *ZmG6PDH1* expression in developing seeds was evident at 25 DAF.

### Analyses of *ZmG6PDH* expression and activity levels under abiotic stress conditions

**Figure 6:**
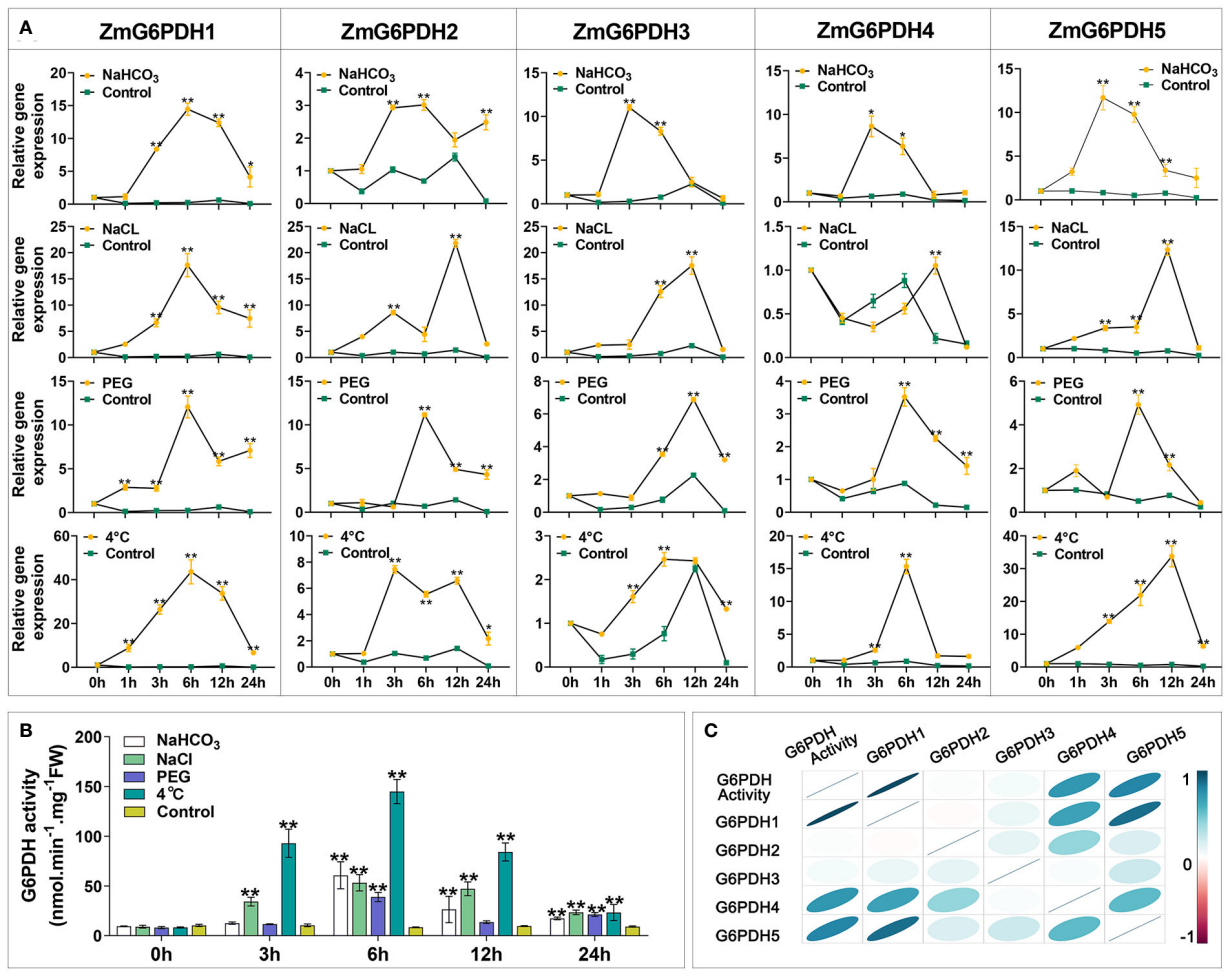
Expression and activity of *ZmG6PDHs* in response to abiotic stressors. **(A)** *ZmG6PDH* expression profiles and **(B)** enzymatic activity levels were measured in maize leaves exposed to 120 mM NaCl, 100 mM NaHCO_3_, 20% PEG, or 4°C conditions for 0, 1, 3, 6, or 12 h *P< 0.05, ** P< 0.01 vs. control; Student’s t-test. **(C)** Correlation coefficients between *ZmG6PDH* expression and G6PDH enzyme activity. Correlations between pairs of traits are shown as individual ellipse charts; colors and slopes indicate the magnitude of correlations. Ellipses corresponding to negative and positive correlations are shown in red and blue, respectively.

Members of the *G6PDH* gene family play vital roles in stress adaptation in various model plants (Yang et al., 2019; Linnenbrügger et al., 2022). *ZmG6PDH* transcriptional profiles were examined in response to low temperature (4°C), alkali (150 mM NaHCO_3_), salt (200 mM NaCl), and drought (20% PEG) stress treatment conditions to explore the potential roles of these genes in maize abiotic stress responses. Alkali treatment resulted in the upregulation of most of these *ZmG6PDHs* (**Figure 6A**), with cytosolic *ZmG6PDHs* exhibiting particularly high transcription levels at 6 h post-stimulation. *ZmG6PDH2, 3*, and *5* upregulation were also observed during the middle stages of salt stress, and at 12 h post-treatment, the *ZmG6PDH2* levels were significantly higher than those of other analyzed genes. All *ZmG6PDHs* other than *ZmG6PDH3* were upregulated at 6 h under osmotic stress conditions, with maximum upregulation at 12 h post-treatment. Cold stress significantly increased *ZmG6PDH* expression; *ZmG6PDH1* was the most cold-inducible gene, reaching maximum expression levels after incubation at 4°C for 6 h (**Figure 6A**).

Measurement of enzyme activity showed that the activities of the G6PDHs in maize plants rose in response to salt, alkali, drought, and osmotic stressors (**Figure 6B**). Roughly 10-15-fold increases in G6PDH activity levels were detected in response to these treatments relative to control conditions, with even more pronounced upregulation being observed in response to cold stress such that enzymatic activity rapidly increased within 6 h of incubation at 4°C (**Figure 6B**). Throughout alkali stress treatment, G6PDH activity levels initially rose to a peak at 12 h and then decreased. Under osmotic stress conditions, G6PDH activities peaked at 6 h and then rose again from 12-24 h. Correlation analyses indicated that G6PDH activity under abiotic stress conditions was consistent with the transcripts of *ZmG6PDH1*, suggesting that it encodes the primary G6PDH isoform involved in cold stress responses (**Figure 6C**).

### Analyses of the effects of *ZmG6PDH1* knockout on abiotic stress responses

**Figure 7:**
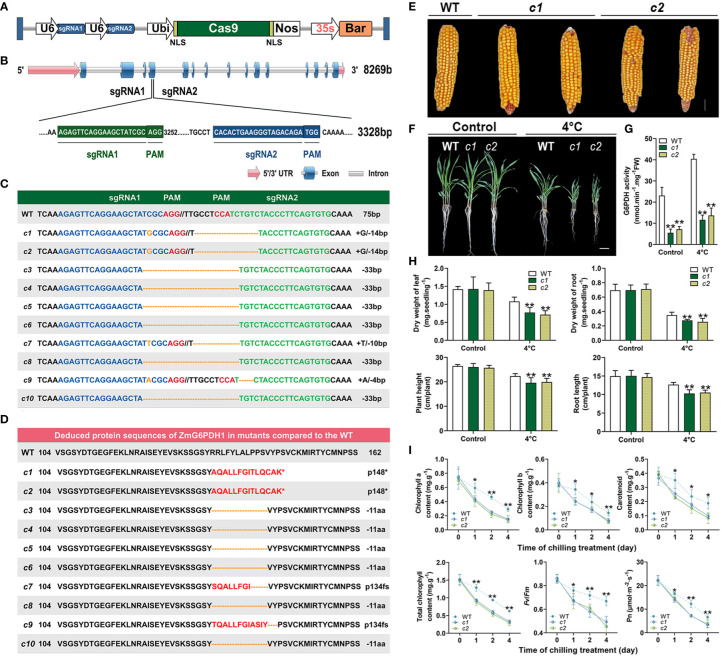
Phenotypic characteristics of *ZmG6PDH1* mutant plants exposed to cold stress. **(A)** Schematic overview of the T-DNA structure of the CRISPR/Cas9 constructs. **(B)** *ZmG6PDH1* gene structure and target sites. **(C)** Gene-edited allele sequences in individual *zmg6pdh1* mutants compared with the WT B73 reference sequence shown above. Red letters denote the PAM, and blue and green indicate target sequences. Deletions and insertions are shown as dashes and orange letters, respectively. Sequence changes relative to the B73 reference genome are annotated on the right. **(D)** Deduced ZmG6PDH1 protein sequences in independent zmg6pdh1 single mutants compared with the B73 reference sequence. An asterisk represents stop codons. **(E)** Mature seed performance comparisons in WT and gene-edited *ZmG6PDH1* mutants. **(F)** The phenotypic characteristics of *zmg6pdh1* mutant plants grown in pots and exposed to cold stress. **(G)** G6PDH enzymatic activity levels and **(H)** dry weight, plant height, and root length were compared in WT and *zmg6pdh1* mutant plants cultivated under control or 4°C treatment conditions. **(I)** Chlorophyll a, chlorophyll b, carotenoid, total chlorophyll, Fv/Fm, and Pn levels in the leaves of WT and *zmg6pdh1* mutant plants following exposure to 4°C conditions for 4 days. Data are means ± SEs (n≥5). *P< 0.05, ** P< 0.01 vs. WT; Student’s t-test.

To fully understand how *ZmG6PDH1* regulates cold stress responses in plants, a CRISPR/Cas9 approach was next used to knock out this gene. Two gRNAs specific for *ZmG6PDH1* (gRNA1 and gRNA2) were cloned into the binary vector p0195 (**Figures 7A, B**) under the control of the maize U6 promoter. The resultant constructs were then introduced into *Agrobacterium tumefaciens*, and maize embryos from the inbred B73 line were transformed *via* Agrobacterium-mediated transformation. In total, 20 T0 plants were collected harboring mutations at the target site, as confirmed through PCR and Sanger sequencing (**Figure S1**). Gene editing occurs at both target sites, making deletions more common than insertions (**Figure 7C**). Large 33 bp deletions were observed between the target sites of sgRNA1 and sgRNA2 (**Figure 7C**). Single nucleotide insertions were the most common type observed at these target sites, each target site, with 40% of insertions being ‘G’ (50%), ‘T’ (25%), or ‘A’ (25%) residues. In total, 8 (40%) of the 20 T0 plants were found to be successfully generated mutants, and 75% and 63% of the mutant T0 plants exhibited homozygous mutations at the respective sgRNA1 and sgRNA2 target sites.

Amino acid sequences for these mutant strains were analyzed, revealing differences in the sequences due to insertions and deletions of varying lengths that contributed to frameshifts and premature translational termination such that the gene was not appropriately expressed (**Figure 7D**). Varying levels of mutation were thus observed in these target genes in the resultant transgenic plants. Measurement of the expression and enzyme activity of *ZmG6PDH1* in the CRISPR-edited maize lines (*c1-c10*) (**Figure S1**) showed that *ZmG6PDH1* levels were 3-10-fold lower than in WT plants (**Figure S1**). Consistently, G6PDH activity in these edited lines was 2-4-fold below that in WT controls (**Figure S1**). These results thus confirmed successful *ZmG6PDH1* knockout in the transgenic plants.

T3 plants generated from the progeny of two editing events (*c1* and *c2*) were used for downstream use. These mutants harbored either a homozygous 1-bp insertion (gRNA1) or a 14-bp deletion (gRNA2), respectively, and these mutations were stably inherited through the T0, T1, and T2 generations as indicated through targeted sequencing analyses. No apparent differences in seeds of visible growth phenotypes (such as plant height, ear height, kernels per ear, or kernels per ear row) were evident when comparing WT and mutant lines under normal growth conditions (**Figure 7E**; **Table S5**). In contrast, upon exposure of 3-week-old soil-grown seedlings to cold stress (4°C) for 4 days, the *ZmG6PDH1*-knockout plants were more sensitive to cold stress than were WT plants, as indicated by decreased height and root elongation, together with lower root and leaf dry weight (**Figures 7F, H**). Significant reductions in G6PDH enzyme activity levels were observed in the *c1* and *c2* lines, whereas they were increased in WT plants under normal and stress conditions (**Figure 7G**), indicating a potential link between *ZmG6PDH1* and G6PDH activity. In addition, mutant plants exhibited lower average total chlorophyll, chlorophyll a, chlorophyll b, and carotenoid levels as compared to WT plants under cold stress conditions (**Figure 7I**), together with a significant drop in the chlorophyll fluorescence parameter (*F_v_*/*F_m_*) and net photosynthetic rate (**Figure 7I**). Together, these results supported a positive role for cytosolic *ZmG6PDH1* as a regulator of maize cold tolerance.

### The impact of *ZmG6PDH1* knockout on cell redox pairs under cold stress conditions

**Figure 8:**
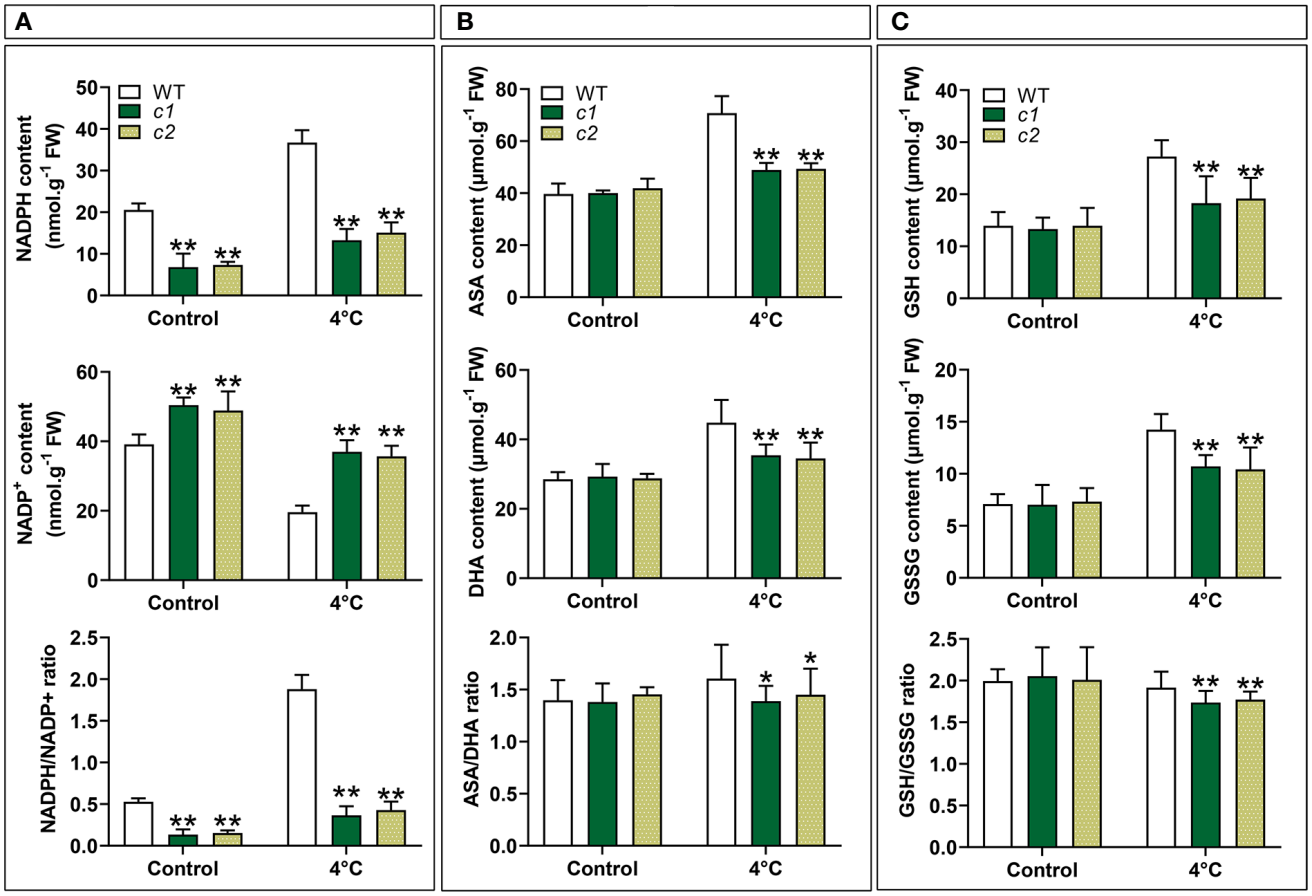
The impact of low-temperature exposure on the redox state of the NADPH, ASA, and GSH pools in WT and *zmg6pdh1* mutant leaves under control and 4°C treatment conditions. **(A)** NADH content, NAD^+^ content, and the NADH/NAD^+^ ratio. **(B)** ASA content, DHA content, and the ASA/DHA ratio. **(C)** GSH content, GSSG content, and the GSH/GSSG ratios. Data are means ± SD (n=3). *P< 0.05, ** P< 0.01 vs. WT; Student’s t-test.

G6PDHs have previously been shown to help maintain proper carbon flow and NADPH generation within the PPP (Chen et al., 2022). NADPH redox status was initially analyzed to confirm the ability of cytosolic *ZmGPDH1* to modulate the redox state within cells under cold stress conditions. Under normal conditions, the NADPH levels and NADPH/NADP^+^ ratios in the *c1* and *c2* lines were reduced relative to WT plants (**Figure 8A**). Increases in the NADPH/NADP^+^ ratios were evident in all mutant lines exposed to cold-stress conditions, and these *zmg6pdh1* mutants exhibited NADH levels persistently lower than those of WT plants, thereby reducing the overall NADH/NAD^+^ ratio despite a pronounced stress-induced increase in NADH levels, suggesting that the cytosolic G6PDH encoded by *ZmG6PDH1* regulates NADPH/NADP^+^ homeostasis (**Figure 8A**).

To more fully explore whether *ZmG6PDH1* gene knockout had any effect on other redox pairs, the redox status of the GSH and ASA pools was also analyzed. No changes in reduced/oxidized GSH and ASA levels were evident under normal growth conditions when comparing WT and mutant plants (**Figures 8B, C**). However, after cold-stress exposure, GSH and ASA levels increased significantly in the WT plants than in the *zmg6pdh1* mutants. These differences coincided with a reduction in the ASA/DHA and GSH/GSSG ratios in *zmg6pdh1* mutants compared with WT plants. Together, these findings indicated that *ZmG6PDH1*–knockout plants could not provide the reducing equivalent NADPH needed for the biosynthesis of ASA and GSH, highlighting a key role for the cytosolic G6PDH encoded by *ZmG6PDH1* as a regulator of the biosynthesis of ASA and GSH under cold-stress conditions.

### The impact of *ZmG6PDH1* knockout on ROS accumulation and antioxidant enzyme levels under low-temperature stress conditions

**Figure 9:**
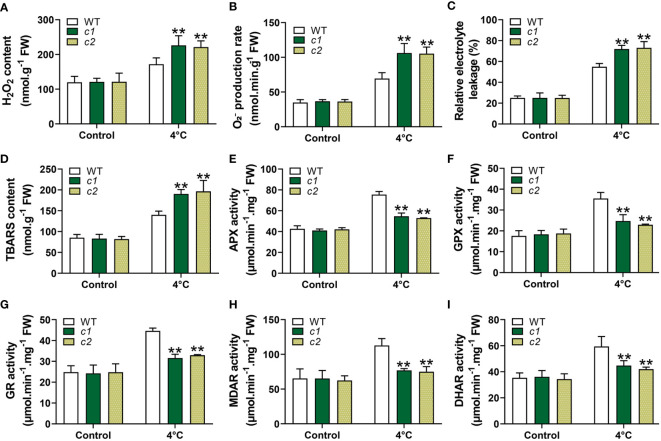
ROS levels and associated antioxidant response activities in WT and *zmg6pdh1* mutant leaves under control or 4°C treatment conditions. **(A)** $O_{2}^{-}$ , **(B)** H_2_O_2_, **(C)** relative electrolyte leakage, and **(D)** TBARS levels were analyzed in WT and *zmg6pdh1* mutants exposed to 4°C conditions. Levels of **(E)** APX, **(F)** GPX, **(G)** GR, **(H)** MDAR, and **(I)** DHAR activities were measured to assess antioxidant activity. FW, fresh weight. Data are means ± SD (n=3). *P< 0.05, ** P< 0.01 vs. WT; Student’s t-test.

The redox state of plants is closely tied to ROS production and processing under stress conditions (Foyer and Noctor, 2005). The reduced NADP(H), ASA, and GSH pools seen in the *zmg6pdh1* mutants thus highlighted a need to assess levels of ROS, including those of hydrogen peroxide (H_2_O_2_) and superoxide radicals $\left(O_{2}^{-}\right)$ , demonstrating that ROS levels were similar in WT and mutant plants under normal conditions. Under cold-stress conditions, however, the levels of ROS in the mutant lines were roughly double those in WT plants (**Figures 9A, B**). TBARS and electrolyte leakage levels related to oxidative damage to the cell membrane were also significantly increased in *c1* and *c2* relative to WT plants exposed to cold stress (**Figures 9C, D**). These data indicate that Zm*G6PDH1* deficiencies contribute to stress-driven ROS accumulation and associated lipid peroxidation. These higher levels of ROS generation can induce the activation of systems responsible for ROS scavenging. Accordingly, levels of activity for antioxidant enzymes, including MDAR, APX, GR, DHAR, and GPX, were assessed. As expected, the activities of these enzymes were more significant in response to cold stress, with lower antioxidant enzyme activity levels seen in the *zmg6pdh1* mutants compared with the WT plants (**Figures 9E-I**). Overall, these findings indicated that the *zmg6pdh1* mutants showed significantly impaired antioxidant and redox systems, consistent with an important role for *ZmG6PDH1* as a modulator of redox homeostasis and ROS scavenging under cold stress conditions.

## Discussion

Maize (*Zea mays* L.) is a major global cereal crop widely used to prepare animal feed, industrial materials, and biofuel (Sheoran et al., 2021). G6PDHs have been identified as important regulators of many plant species’ growth and abiotic stress responses (Wakao and Benning, 2005; Landi et al., 2021; Tian et al., 2021). While *G6PDHs* have been cloned successfully from a range of plants, including soybean (Zhao et al., 2020), tobacco (Yang et al., 2022), tomato (Landi et al., 2016), barley (Cardi et al., 2013), wheat (Nemoto and Sasakuma, 2000), and *Arabidopsis* (Wakao and Benning, 2005), little is known regarding this gene family in maize. Five maize *G6PDH* family members were identified (*ZmG6PDH1-5*; **Table 1**). Much as has been reported for other G6PDHs (Landi et al., 2021), the identified ZmG6PDHs contained key conserved protein domains (PF00479, PF02781) (**Figure 1B**). All five of these proteins expressed the conserved NEFVIRLQP motif (**Figure S1**), as has been reported for NADP^+^-dependent G6PDH isoforms with analogous NADP^+^-binding fragments corresponding to NEFVIRLQP (Yang et al., 2014; Zhang et al., 2020). The presence of signal peptides determines the localization of G6PDHs within plant cells, and transient expression of GFP-tagged versions of these ZmG6PDHs in maize mesophyll protoplasts was performed to evaluate their subcellular localization (**Figure 4**), demonstrating that ZmG6PDH2, 3, and 4 fusion proteins localized to the chloroplast compartment whereas ZmG6PDH1 and 5 localized to the cytosol (**Figure 4**). These findings were consistent with prior *in silico* predictions and the phylogenetic clades to which these ZmG6PDHs were assigned. Prior studies hypothesized that AtG6PDHs would localize to the plastid or cytosolic compartments based on their targeting signals and transmembrane domains, but there was a lack of experimental evidence to support these predictions (Wakao and Benning, 2005; Landi et al., 2021).

G6PDH family enzymes reportedly play essential roles in the biosynthesis of lipids during plant seed development (Yang et al., 2019). Here, *ZmG6PDH* expression was observed in all analyzed tissues, with these levels being particularly high in leaves, tassels, and developing seeds (**Figure 5**). This is consistent with earlier results on *Arabidopsis* and soybean, with high levels of the *AtG6PDHcy* isoform detected in developing siliques (Yang et al., 2019) and high expression of soybean *G6PDH*s observed during seed development (Zhao et al., 2020). These tissue-specific expression patterns suggest key physiological roles for these *ZmG6PDHs* as regulators of maize development (**Figure 5**). Further physiological analyses were thus conducted to examine the *ZmG6PDH*-mediated regulation of abiotic stress adaptation. More NADPH is required to maintain a normal redox state in plants under abiotic stress (Yang et al., 2014). As indicated by our results, this may increase G6PDH. The *ZmG6PDHs* were significantly upregulated in response to salt, alkali, osmotic, and drought stress (**Figures 6A, B** **)**, in line with the *AtG6PDH* (Wakao and Benning, 2005), *HbG6PDH* (Long et al., 2016), *PsG6PDH* (Lin et al., 2005) and *ScG6PDH* (Begcy et al., 2012; Yang et al., 2014) activity and expression patterns reported previously. Notably, a cytosolic isoform (*ZmG6PDH1*) responds vigorously to cold stress (**Figure 6C**), suggesting it is an important regulator of these cold stress responses. Similar results have been observed in poplar (*Populus suaveolens*) and sugarcane (*Saccharum officinarum*): a cytosolic *G6PDH* from *Populus suaveolens* was identified as an important mediator of enhanced cold resistance in tobacco plants (Lin et al., 2005); and a cytosolic *ScG6PDH* in sugarcane also played a positive role in response to cold stress (Yang et al., 2014), while the associated functional verification is required.

Two homozygous *ZmG6PDH1* mutants generated using a CRISPR/Cas9 approach were isolated to confirm these results further. Morphologically, these *ZmG6PDH1* knockout mutants appeared comparable to WT plants (**Figure 7**). However, both mutant strains showed increased sensitivity to cold stress seen in the reductions in fresh weight, height, and root length after cold exposure (**Figure 7**). Cytosolic *ZmG6PDH1* deficiency may thus influence the ability of plants to adapt to cold conditions and may be capable of exacerbating growth suppression under cold temperatures. Cytosolic G6PDHs have been shown to supply NADPH and thus modulate cells’ redox status (Valderrama et al., 2006). Consistently, the *zmg6pdh1* mutants in this study showed increased NADP^+^ levels and reduced NADPH formation compared with WT plants (**Figures 8A**), with a corresponding drop in the cellular NADPH/NADP^+^ ratio (**Figures 8A**), indicating that the impaired metabolic activity in these plants had profoundly compromised NADPH generation. Evident decreases in ASA/DHA and GSH/GSSG levels were also evident in both *zmg6pdh1* mutants as compared to WT plants (**Figure 8B**), indicating that the loss of *ZmG6PDH1* affected the redox status of the ASA pool beyond the immediate increase in this NADPH/NADP^+^ ratio. Overall, these findings highlighted the critical role that *ZmG6PDH1* plays as a modulator of the cellular redox homeostasis of the GSH, ASA, and NADP(H) pools under cold stress conditions, in line with prior evidence (Wang et al., 2016).

*ZmG6PDH1* deficiency also resulted in increases in ROS, including chloroplastic ROS (**Figures 9A, B** **)**, seen by the significantly reduced chlorophyll content in *zmg6pdh1* mutant plants exposed to cold stress (**Figures 7I**). Membrane damage in *zmg6pdh1* mutant plants was more severe, shown by the greater TBARS content and electrolyte leakage under stress conditions (**Figures 9C, D** **)**, emphasizing that knockout of *ZmG6PDH1* led to significant ROS accumulation and associated increases in lipid peroxidation. ASA and GSH are non-enzymatic antioxidant members of the ASA-GSH cycle responsible for their regeneration, enabling them to eliminate excess ROS within cells (Noctor and Foyer, 1998). *ZmG6PDH1* may thus regulate the ASA-GSH cycle to influence ROS metabolism.

Enzymatic antioxidants in the ASA-GSH cycle play a key role in maintaining redox balance within cells and include APX, GR, GPX, MDAR, and DHAR (Noctor and Foyer, 1998; Foyer and Noctor, 2005). GR maintains a robust GSH pool within cells that is required to ensure active protein functionality, as it can prevent non-specific mixed disulfide bond formation and the consequent aggregation or inactivation of proteins (Couto et al., 2016). APX can utilize ASA as an electron donor for H_2_O_2_ scavenging, oxidizing it to yield MDHA (Gill and Tuteja, 2010). GPX is the key enzyme responsible for repairing lipid peroxidation and an essential enzymatic mediator of antioxidant protection against membrane damage (Foyer and Noctor, 2005). Reduced GSH and DHA act as substrates of DHAR, a key enzyme for reduced ASA. MDAR utilizes NADPH as an electron donor to catalyze the processing of monodehydroascorbate (MDHA) into DHA and ASA (Singh et al., 2015). Here, cold treatment was found to strongly induce APX, GR, GPX, MDAR, and DHAR activity in WT plants (**Figures 9E** **-**I), resulting in higher levels of GSH and ASA accumulation and decreases in ROS levels, whereas the same was not observed in *zmg6pdh1* mutants. Significant decreases in GSH, ASA, and NADPH levels, together with reductions in the activities of these key enzymes in the *zmg6pdh1* mutant plants, highlight the key role that *ZmG6PDH1* plays as a modulator of the ASA-GSH redox cycle, providing the NADPH necessary for the biosynthesis of GSH and ASA. Redox signaling plays an essential role in inter-organizational communication and nuclear gene expression regulation (Wolin et al., 2007; Kopczewski and Kuźniak, 2013). The low levels of NADPH in *zmg6pdh1* mutants were related to the impairment of the oxidative pentose phosphate pathway. Changes in the metabolic redox state (NADP^+^/NADPH ratio) can represent a sensor for environmental fluctuation and serve as signals that coordinate the nuclear gene expression with the physiological response to cold stress. Together, these data demonstrate the important role of *ZmG6PDH1* as a regulator of cold tolerance through its ability to influence the cellular redox state and ROS-scavenging system, thus helping to balance ROS generation and to alleviate associated cellular toxicity.

## Conclusion

In conclusion, five *G6PDH* genes encoded by *Z. mays* were systematically identified and characterized. Phylogenetic and subcellular localization analyses enabled the classification of these *ZmG6PDHs* into cytosolic and plastidic isoforms. The expression of these different *ZmG6PDH* family members varied in response to particular abiotic stressors underscoring the distinct regulatory roles played likely by these enzymes. Cytosolic *ZmG6PDH1* expression was responsive to cold stress exposure and highly correlated with G6PDH activity levels, indicating that it is likely to play a key role in cold stress responses. Following this gene’s CRISPR/Cas9-mediated knockout, *zmg6pdh1* mutant seedlings exhibited increased cold stress sensitivity compared with WT seedlings. Further research indicated that this gene encodes an active G6PDH enzyme form that maintains ASA and GSH redox homeostasis to mitigate oxidative damage induced by cold exposure.

## Data availability statement

The datasets presented in this study can be found in online repositories. The names of the repository/repositories and accession number(s) can be found in the article/**supplementary material**.

## Author contributions

XL, QC, and TY designed and conceived the experiments. XL performed the experiments. XL, ShL, SiL, YL, YS, HR, and JJZ analyzed the data and interpreted the results. XL prepared the manuscript. YZ, JGZ, and YHZ conceived the experiments and revised the manuscript. All authors agreed to be accountable for all aspects of the work, ensuring that questions related to the accuracy or integrity of any part of the work are appropriately investigated and resolved, and approved the final version to be published. All authors contributed to the article and approved the submitted version.
