# Supplementary material for: ZmG6PDH1 in glucose-6-phosphate dehydrogenase family enhances cold stress tolerance in maize
Source: Front Plant Sci. 2023 Mar 9;14:1116237. doi: 10.3389/fpls.2023.1116237 (PMC10034328; doi:10.3389/fpls.2023.1116237)
Supplement: Supplementary file 1 [file DataSheet_1.docx]

**Supplementary Material**

**Table S1.** The gene ID and location of *G6PDH* genes used in this study.

| **Gene Name** | **Gene ID** | **Gene Location** | | |
| --- | --- | --- | --- | --- |
|  |  | **Chromosome** | **Start** | **End** |
| *ZmG6PDH1* | GRMZM2G130230_T01 | Zm2 | 37085168 | 37093582 |
| *ZmG6PDH2* | GRMZM2G177077_T01 | Zm10 | 99381158 | 99387758 |
| *ZmG6PDH3* | GRMZM2G426964_T01 | Zm9 | 134182737 | 134185295 |
| *ZmG6PDH4* | GRMZM2G179521_T01 | Zm1 | 72102454 | 72121802 |
| *ZmG6PDH5* | GRMZM2G031107_T02 | Zm5 | 181440754 | 181446379 |
| *SbG6PDH1* | Sobic.001G339100.1 | Sb1 | 62704645 | 62707868 |
| *SbG6PDH2* | Sobic.006G030800.1 | Sb6 | 6518696 | 6524700 |
| *SbG6PDH3* | Sobic.004G204900.1 | Sb4 | 55632561 | 55639949 |
| *SbG6PDH4* | Sobic.006G126300.1 | Sb6 | 49163049 | 49169784 |
| *SbG6PDH5* | Sobic.001G390900.2 | Sb1 | 67738026 | 67743372 |
| *AtG6PDH1* | AT5G35790.1 | At5 | 13956690 | 13959753 |
| *AtG6PDH2* | AT5G13110.1 | At5 | 4158811 | 4161820 |
| *AtG6PDH3* | AT1G24280.1 | At1 | 8609445 | 8612580 |
| *AtG6PDH4* | AT1G09420.2 | At1 | 3038469 | 3041873 |
| *AtG6PDH5* | AT3G27300.1 | At3 | 10083049 | 10086696 |
| *AtG6PDH6* | AT5G40760.1 | At5 | 16310748 | 16314774 |
| *PvG6PDH1* | Phvul.010G143200.1 | Pv10 | 42525909 | 42530526 |
| *PvG6PDH2* | Phvul.008G148700.1 | Pv8 | 42532948 | 42540782 |
| *PvG6PDH3* | Phvul.004G057000.1 | Pv4 | 7865449 | 7880983 |
| *PvG6PDH4* | Phvul.001G223300.1 | Pv1 | 47839745 | 47845231 |
| *PvG6PDH5* | Phvul.008G016900.1 | Pv8 | 1372055 | 1376747 |
| *BolG6PDH1* | Bol041759 | Bol07 | 16063908 | 16066648 |
| *BolG6PDH2* | Bol014038 | Bol01 | 18945387 | 18945387 |
| *BolG6PDH3* | Bol005284 | Bol03 | 43146340 | 43147737 |
| *BolG6PDH4* | Bol022125 | Bol08 | 17363663 | 17366624 |
| *BolG6PDH5* | Bol042879 | Bol07 | 32749457 | 32752756 |
| *TaG6PDH1* | Traes_2AS_67222868A.1 | Ta2AS | 654 | 5570 |
| *TaG6PDH2* | Traes_6AL_BFF9BD51D.1 | Ta6AL | 3580 | 9469 |
| *TaG6PDH3* | Traes_6DS_C6B1E3EE7.1 | Ta6DS | 5257 | 8318 |
| *TaG6PDH4* | Traes_2DL_06E6A2543.1 | Ta2DL | 2007 | 8781 |
| *TaG6PDH5* | Traes_6AS_62C3F99C5.1 | Ta6AS | 3184 | 6146 |
| *SiG6PDH1* | Seita.9G424300.1 | Si9 | 47991389 | 47996499 |
| *SiG6PDH2* | Seita.4G038800.1 | Si4 | 2605838 | 2606128 |
| *SiG6PDH3* | Seita.7G143800.1 | Si7 | 23280005 | 23286389 |
| *SiG6PDH4* | Seita.7G029700.1 | Si7 | 8314258 | 8320650 |
| *SiG6PDH5* | Seita.1G221400.1 | Si1 | 30020405 | 30026817 |
| *SlG6PDH1* | Solyc01g100960.2 | Sl1 | 90837020 | 90843494 |
| *SlG6PDH2* | Solyc07g045540.2 | Sl7 | 58675918 | 58681483 |
| *SlG6PDH3* | Solyc05g015950.2 | Sl5 | 13085211 | 13089624 |
| *SlG6PDH4* | Solyc02g093830.2 | Sl2 | 54535933 | 54541841 |
| *StG6PDH1* | Soltu.DM.05G012290.1 | St5 | 15880052 | 15884736 |
| *StG6PDH2* | Soltu.DM.02G033900.6 | St2 | 45241377 | 45248271 |
| *StG6PDH3* | Soltu.DM.01G040700.1 | St1 | 79225797 | 79233440 |
| *StG6PDH4* | Soltu.DM.07G015360.1 | St7 | 45211539 | 45217294 |

**Table S2.** The primers used in this study.

| **Primer Names** | **Sequence (5'-3')** | **Description** |
| --- | --- | --- |
| ZmG6PDH1-F | ATGTCAGGAGGATCTTCACCATCA | cloning |
| ZmG6PDH1-R | CTATGCAAGGGTCGGCGG | cloning |
| ZmG6PDH2-F | ATGGCGCTCTCCTGCATGA | cloning |
| ZmG6PDH2-R | TCATGCCTTGTAGTGCTCCGC | cloning |
| ZmG6PDH3-F | ATGCCATGGGAACAACCTC | cloning |
| ZmG6PDH3-R | TCACCAGTCGTCATCCCATCG | cloning |
| ZmG6PDH4-F | ATGTCCATTCGACCATTCCTGA | cloning |
| ZmG6PDH4-R | CTAGAAGGCGCCGTCGTCA | cloning |
| ZmG6PDH5-F | ATGTCAGGAGGGTCGTCTGAATC | cloning |
| ZmG6PDH5-R | CTAAACCTTAGAAAGGGTCGGCG | cloning |
| ZmG6PDH1-qF | TCACTCCTTTGCTGCATGAC | RT-qPCR |
| ZmG6PDH1-qR | GGGTCTGCACGTATCCAACT | RT-qPCR |
| ZmG6PDH2-qF | CCTACCTCACCAAAGGTGGA | RT-qPCR |
| ZmG6PDH2-qR | AAATGACATGCCTTGGGAAG | RT-qPCR |
| ZmG6PDH3-qF | CACCCAAACAGGCGTATTCT | RT-qPCR |
| ZmG6PDH3-qR | CCAGGCTTGCAAGTTTTAGC | RT-qPCR |
| ZmG6PDH4-qF | AACGACCAAGAGCTCAGGAA | RT-qPCR |
| ZmG6PDH4-qR | CTGCCCAGACTGGTAGAAGC | RT-qPCR |
| ZmG6PDH5-qF | GTTCTCCGCTCTTGGTCTTG | RT-qPCR |
| ZmG6PDH5-qR | TAACGGGGAAGATTCAGACG | RT-qPCR |
| ZmACTIN-qF | ATCCAGGCTGTTCTTTCGTT | RT-qPCR |
| ZmACTIN-qR | CATTAGGTGGTCGGTGAGGT | RT-qPCR |
| ZmGAPDH-qF | CCCTTCATCACCACGGACTAC | RT-qPCR |
| ZmGAPDH-qR | AACCTTCTTGGCACCACCCT | RT-qPCR |
| ZmG6PDH1-F | AGTGGTTCCTATGATACTGG | Genotyping |
| ZmG6PDH1-R | ACTCCACCATACTTGTTTGT | Genotyping |

**Table S3.** Specific gRNAs targeting *ZmG6PDH1*.

| **sgRNA** | **Sequence (5'-3')** | **PAM (5'-3')** | **Orientation** |
| --- | --- | --- | --- |
| gRNA1 | AGAGTTCAGGAAGCTATCGC | AGG | **+** |
| gRNA2 | CACACTGAAGGGTAGACAGA | TGG | **-** |

| **Table S4.** The syntenic relationships among *G6PDH* genes. | | | | | | | | | | |
| --- | --- | --- | --- | --- | --- | --- | --- | --- | --- | --- |
| **Block** | ***Zea mays gene location*** | | | ***Zea mays gene* ID** | ***Zea mays* gene name** | ***Zea mays gene location*** | | | ***Zea mays gene* ID** | ***Zea mays* gene name** |
|  | **Chr** | **start** | **end** |  |  | **Chr** | **start** | **end** |  |  |
| 242 | Zm5 | 181440754 | 181446379 | GRMZM2G031107_T02 | *ZmG6PDH5* | Zm2 | 37085168 | 37093582 | GRMZM2G130230_T01 | *ZmG6PDH1* |
| **Block** | ***Arabidopsis thaliana* gene location** | | | ***Arabidopsis thaliana* gene ID** | ***Arabidopsis thaliana***  **gene name** | ***Zea mays* gene location** | | | ***Zea mays* gene ID** | ***Zea mays* gene name** |
|  | **Chr** | **start** | **end** |  |  | **Chr** | **start** | **end** |  |  |
| 247 | At3 | 10083048 | 10086696 | AT3G27300.1 | *AtG6PDH5* | At5 | 16310748 | 16314774 | AT5G40760.1 | *AtG6PDH6* |

**Table S5.** Comparison of growth traits between *ZmG6PDH1* mutants and WT plants. Means ± s.d. (n=10).

| **Strains** | **Plant height (cm)** | **Ear height**  **(cm)** | **Number of kernels per ear** | **Number of kernels in each row of the ear** | **Kernel length per plant (cm)** | **Kernel width per plant (cm)** | **Kernel thickness**  **per plant (cm)** |  |
| --- | --- | --- | --- | --- | --- | --- | --- | --- |
| WT | 192.3 ± 11.3 | 78.2 ± 4.60 | 16.80 ± 2.01 | 39.20 ± 2.41 | 0.98 ± 0.10 | 1.02 ± 0.11 | 0.42 ± 0.02 |  |
| *C1* | 188.4 ± 9.1 | 77.5 ± 3.61 | 17.10 ± 1.12 | 40.30 ± 3.32 | 0.97 ± 0.09 | 1.01 ± 0.02 | 0.41 ± 0.02 |  |
| *C2* | 194.1 ± 12.6 | 77.5 ± 5.23 | 16.60 ± 1.73 | 38.50 ± 2.30 | 0.98 ± 0.11 | 1.01 ± 0.17 | 0.40 ± 0.07 |  |
| Note: The data represent the means ± SD of five replicate experiments and were analyzed by unpaired two-tailed Student’s *t*-test. * Significant increase (p < 0.05) compared with WT. ** Extremely significant increase (p < 0.01) compared with WT. | | | | | | | |  |
|  |  |  |  |  |  |  |  |  |


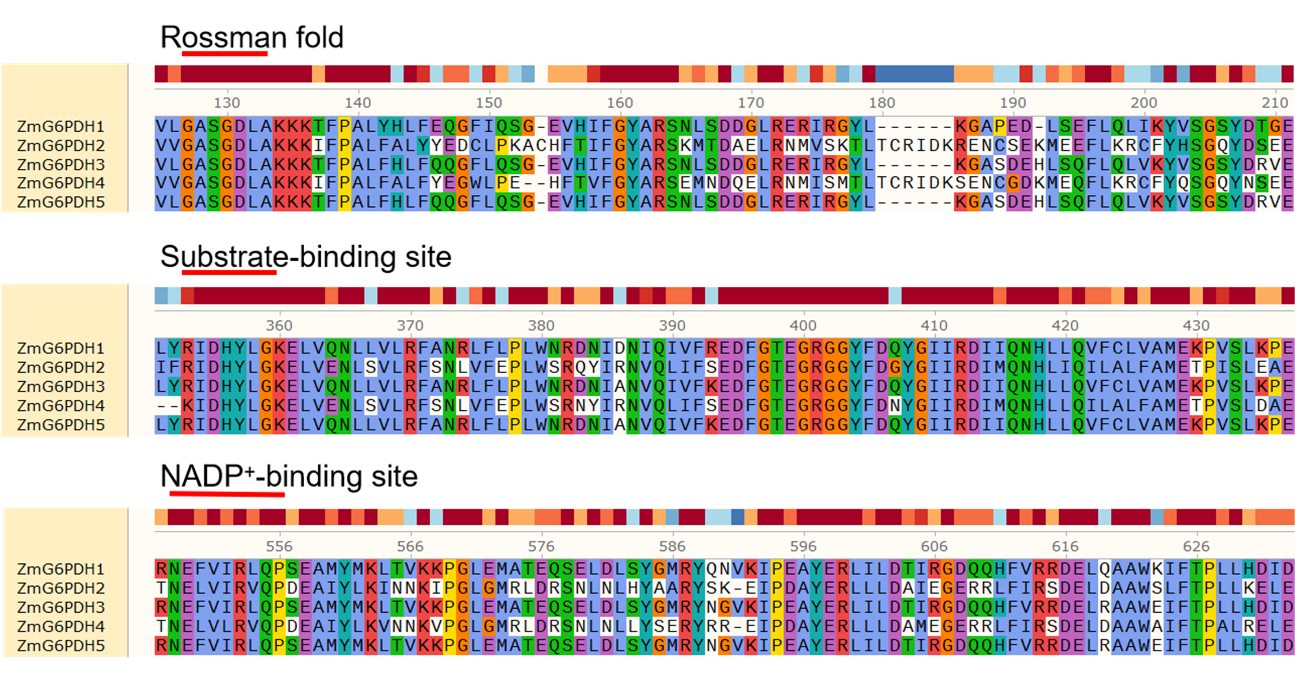


**Figure S1.** Multiple alignment of protein sequences of soybean G6PDHs.


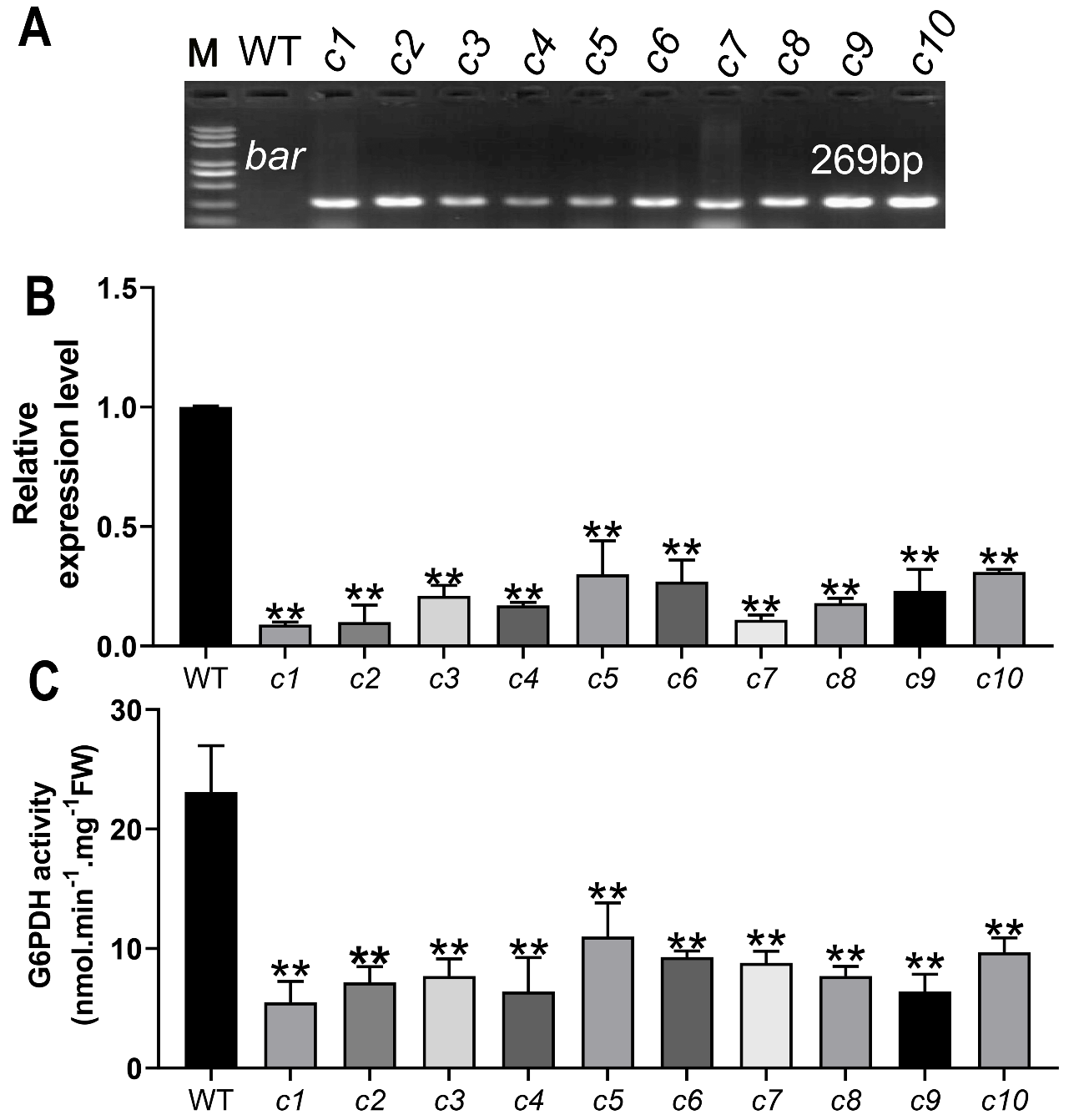


**Figure S2.** **(A)** PCR analysis of the WT and CRISPR-edited maize lines (*c1-c10*). M: DNA marker; WT, the wild type B73. **(B)** Gene expression and **(C)** enzyme activities of *ZmG6PDH1* in WT and CRISPR-edited maize lines (*c1-c10*). Asterisks indicated significant differences from the WT, as determined by Student′s t-test (*P<0.05, ** P<0.01).
